# Fu’s subcutaneous needling facilitates muscle repair by regulating mitochondrial homeostasis in rat with chronic peripheral nervous pain

**DOI:** 10.3389/fphys.2025.1640735

**Published:** 2025-08-21

**Authors:** Po-En Chiu, Zhonghua Fu, Hung-Chuan Pan, Yi-Ching Tsai, Chia-Yun Tsai, Wei-Jen Hsu, Li-Wei Chou, De-Wei Lai

**Affiliations:** ^1^ Department of Chinese Medicine, Chang Bing Show Chwan Memorial Hospital, Changhua, Taiwan; ^2^ Graduate Institute of Integrated Medicine, College of Chinese Medicine, China Medical University, Taichung, Taiwan; ^3^ Institute of Fu’s Subcutaneous Needling, Beijing University of Chinese Medicine, Beijing, China; ^4^ Clinical Medical College of Acupuncture and Moxibustion and Rehabilitation, Guangzhou University of Chinese Medicine, Guangzhou, China; ^5^ Neurological Institute, Taichung Veterans General Hospital, Taichung, Taiwan; ^6^ Department of Medical Research, Taichung Veterans General Hospital, Taichung, Taiwan; ^7^ Ph.D. Program in Translational Medicine, Rong Hsing Research Center for Translational Medicine, National Chung Hsing University, Taichung, Taiwan; ^8^ Doctoral Program in Biotechnology Industrial Management and Innovation, National Chung Hsing University, Taichung, Taiwan; ^9^ Precision gene and cell center, Chang Bing Show Chwan Memorial Hospital, Changhua, Taiwan; ^10^ Department of Physical Medicine and Rehabilitation, China Medical University Hospital, Taichung, Taiwan; ^11^ Department of Physical Therapy and Graduate Institute of Rehabilitation Science, China Medical University, Taichung, Taiwan; ^12^ Department of Physical Medicine and Rehabilitation, Asia University Hospital, Asia University, Taichung, Taiwan; ^13^ Department of Health-Business Administration, Hungkuang University, Taichung, Taiwan

**Keywords:** Fu’s subcutaneous needling, chronic constriction injury, vastus lateralis, gastrocnemius muscles, mitophagy

## Abstract

Sciatica, often resulting from lumbar disc herniation or nerve compression, disrupts electrical signal transmission, leading to muscle atrophy, mitochondrial dysfunction, and impaired energy metabolism. This study explored the therapeutic effects of Fu’s subcutaneous needling (FSN) in a chronic constriction injury (CCI) rat model, assessing its impact on neuropathic pain, muscle mass, and structural integrity. Histological and ultrastructural analyses demonstrated that FSN alleviated hypersensitivity, reduced muscle atrophy, preserved mitochondrial density, and maintained glycogen storage. Gene expression and pathway enrichment analyses revealed FSN’s involvement in PI3K–Akt, MAPK signaling, oxidative phosphorylation, and mitophagy, suggesting its role in modulating energy metabolism and cellular repair. FSN also normalized energy-related proteins FGFR1, FGFR3 and phosphorylated FOXO3, highlighting their significance in muscle repair and regeneration. These findings provide novel insights into FSN’s potential for counteracting neuropathy-induced muscle damage and improving mitochondrial function, supporting its clinical application. Additionally, FSN’s role in muscle repair suggests a connection between growth factor signaling and nerve regeneration, offering a foundation for future research on muscle-neural recovery mechanisms.

## 1 Introduction

Sciatica is typically caused by lumbar disc herniation, nerve compression (sedentary lifestyle), or other nerve injuries (Qiu et al., 2024). Nerve compression impairs the transmission of electrical signals, potentially leading to the atrophy of associated muscles including the triceps surae and gluteus maximus (Dopke et al., 2024; Petrella et al., 2024). Nerve injury-induced inflammatory responses may induce abnormal muscle cell metabolism, further accelerating the progression of muscle atrophy (Zha, 2025). The current treatment methods include anti-inflammatory drugs, neuromodulators, and muscle relaxants combined with physical therapy (Rasmussen-Barr et al., 2016). Surgical, pulsed radiofrequency or other interventional treatments are used to treat severe nerve compression (Li H. et al., 2024). However, treatment outcomes are often inconsistent, and complete recovery of muscle function remains challenging. Fu’s subcutaneous needling (FSN) therapy effectively alleviates sciatica pain, but evidence supporting its effect on muscle recovery remains limited.

Chronic constriction injury (CCI) is a widely used animal model for investigating compressive neuropathy that elucidates the pathophysiological mechanisms of neuropathic pain and associated muscle atrophy (Baron, 2006; Bennett and Xie, 1988; Ho and Mo, 1992; Leng et al., 2012). Neuropathic pain, a hallmark feature of CCI, is characterized by spontaneous and evoked pain resulting from maladaptive changes in the nervous system (Finnerup et al., 2021). In a CCI model, persistent mechanical nerve compression induced neuroinflammation, demyelination, and Wallerian degeneration, all of which collectively contribute to the onset and maintenance of neuropathic pain (Du et al., 2023; George et al., 2004; Ramer et al., 1997). These pathological processes are accompanied by the upregulation of pro-inflammatory cytokines, such as tumor necrosis factor-α and interleukin-1β, and macrophage infiltration at the injury site, further exacerbating nerve damage (Leung and Cahill, 2010). Moreover, chronic nerve injuries lead to profound metabolic changes and structural alterations in skeletal muscles. CCI-induced sciatic nerve injury increased the levels of intramuscular glucose and polyol pathway intermediates, indicating metabolic dysregulation. These changes are associated with reduced activities of key enzymes involved in glycolysis and glycogen synthesis (Langer et al., 2020). Furthermore, CCI causes significant muscle atrophy and metabolic disruptions, as evidenced by alterations in autophagy markers and the ubiquitin–proteasome system. These effects are partially reversible following nerve release, with improvements in muscle mass and metabolic function (Bosco et al., 2023). These findings underscore the dual impact of chronic nerve injury on muscle structure and metabolism, and highlight the potential of therapeutic interventions to mitigate or reverse these changes. Mitochondrial dysfunction is a key factor in CCI-induced muscle atrophy. Mitochondria are central to cellular energy metabolism, and their structural integrity is critical for maintaining muscle function. From a molecular mechanism perspective, HSP60 ensures mitochondrial protein quality, if dysfunctional or misfolded proteins can signal stress, which may activate pathways like PINK1-Parkin (PARK2)-mediated mitophagy, PINK1 detects damage and recruits Parkin to tag mitochondria via VDAC ubiquitination, and BNIP3L facilitates their selective clearance through autophagy, forming a coordinated mitophagy pathway (De et al., 2021; Jadiya and Tomar, 2020). CCI induces mitochondrial morphological alterations, such as swelling, disrupted cristae, and changes in mitochondrial DNA (mtDNA) content, all of which contribute to muscle weakness and degeneration (Wang et al., 2019). Targeting mitochondrial dysfunction is a promising therapeutic strategy for mitigating muscle atrophy and facilitating recovery.

FSN can alleviate musculoskeletal pain and enhance tissue repair (Chiu et al., 2025; Lin et al., 2020). It involves the insertion of specially designed needles into subcutaneous tissue, followed by horizontal manipulation to achieve therapeutic effects. Unlike traditional acupuncture, FSN often targets areas distal to the primary pain site (Chiu et al., 2022; Li H. Z. et al., 2024). This procedure may induce changes in the membrane potential within the local muscle and fascial layers, influencing the propagation of electrophysiological signals. Through mechanical stimulation, FSN indirectly triggers the transmission of electrical neural signals. Although the induced effects may be more subtle than those of direct electrical stimulation, FSN exerts a pronounced effect on myofascial functional adjustment (Li et al., 2022). Clinical studies have reported significant improvements in pain relief and functional recovery in patients with neuropathic pain following acupuncture interventions (Vickers et al., 2018). When combined with standard medical care, acupuncture is superior to standard care alone in terms of pain management and functional restoration (Liang et al., 2024). Moreover, acupuncture has been associated with enhanced efficacy of conventional treatments, reduced reliance on analgesics, and improved patient satisfaction (Lin et al., 2022). Emerging research has highlighted FSN’s potential to reduce inflammatory responses and promotes axonal regeneration and remyelination in nerve injury animal models (Chiu et al., 2023a; Chiu et al., 2024). FSN may modulate the expression of mitochondrial-related genes, thereby promoting nerve and muscle repair. However, the mechanisms by which FSN regulates mitochondrial function and facilitates tissue repair remain unclear. FSN treatment alleviated CCI-induced neuropathic pain and muscle atrophy by regulating mitochondria-related genes and mitigating mitochondrial autophagy, providing new theoretical and experimental support for its clinical application.

## 2 Materials and methods

### 2.1 Experimental animals and groups

Twenty-four adult Sprague–Dawley rats (200–250 g, 8–10 weeks old) were acclimatized and isolated, housed in ventilated rooms with controlled humidity and temperature (22°C–24°C), under a normal 12-h light/dark cycle, with free access to food and water. All experiments were approved by the Animal Care and Use Committee of the Chang Bing Show Chwan Memorial Hospital (IACUC approved number: #111031).The rats were randomly divided into the sham, CCI, and CCI + FSN groups (n = 8 for each group).

The CCI model was established by modifying the method developed by Bennett et al., in 1988 for chronic constriction injury (CCI) of the sciatic nerve. The rats were anesthetized under 4% isoflurane induction, followed by maintenance with 1%–2% isoflurane. The right sciatic nerve was carefully exposed and tied using 3–0 chromic catgut sutures. The distance between the sutures was approximately 1 mm, and the tightness of all the ligatures was the same. Finally, after ligation, the nerve was returned to its original position, and the muscle and skin layers were sutured. The sham operations involved the exposure of the sciatic nerve without ligation. Pain thresholds were measured on days 1, 3, 5, and 7 after modelling. The presence of neuropathic pain indicated successful modeling, and rats that did not develop neuropathic pain were excluded. The intervention began on the first day after successful modeling (day 8 of the total timeline). The FSN group received FSN needle treatment at fixed time points on days 8, 10, 12, and 14. The treatment frequency and timing of FSN administration were determined based on both our previous clinical and preclinical studies (Chiu et al., 2023a; Chiu et al., 2022; Chiu et al., 2023b; Chiu et al., 2024). On day 14, all animals were euthanized under deep anesthesia. Skeletal muscles from the ipsilateral side—including the vastus lateralis, biceps femoris, and gastrocnemius—were carefully dissected for further analysis. These specific muscles were selected based on their anatomical relationship with the sciatic nerve and were used in subsequent gross morphology assessment, muscle weight quantification, and histological or molecular evaluations.

### 2.2 Animal groups and sciatic nerve surgery

The 8–10-week-old Sprague–Dawley rats were acclimatized, quarantined, and kept in ventilated humidity- and temperature-controlled rooms with a 12-h light/dark cycle. All experiments were approved by the Animal Care and Use Committee of the Chang Bing Show Chwan Memorial Hospital (IACUC approved number: #111031). Twenty-four animals were randomly assigned to three groups: sham, CCI and CCI + FSN. The CCI model was established in a manner similar to the method described (Chiu et al., 2024). Briefly, the right sciatic nerve was loosely ligated using a 3–0 chromic gut. A sham surgery was performed by exposing the sciatic nerve without ligation. All surgical procedures were performed under anesthesia with 4% isoflurane for induction and 1%–2% isoflurane for maintenance.

### 2.3 FSN

Disposable needles were used in the FSN group (Nanjing Paifu Medical Science and Technology Co., Nanjing, China). Without anesthesia, the rat hind limb was extended to tension and the FSN needle was inserted towards the tightened muscle (muscle with Myofascial Trigger Points, approximately near the biceps femoris muscle). The needle was rapidly advanced to prevent tension. After the needle entered the skin, the swaying movement began. This movement is a smooth, gentle, and fan-shaped swaying using the thumb as the pivot. The index, middle, and ring fingers were aligned in straight lines. The middle and thumb faced each other during insertion, whereas the index and ring fingers alternated back and forth. The frequency of this movement was 100 times per minute, with each session lasting approximately 1 min. The procedure was performed every 2 days for a total of four sessions (on days 8, 10, 12, and 14).

### 2.4 Assessment of mechanical allodynia

The rats were placed in a box on a metal mesh, with eight rats per group. They were left to acclimate for 15–30 min until they remained calm in an unfamiliar environment. Each rat was then placed individually in a small cage with a framework and allowed to acclimatize for 5 min before testing began. Von Frey filaments were applied to the ipsilateral hindpaw until it bent for 5 s. The range of the filaments used was 0.008–300 g. A positive response was considered if the animal withdrew or shook its hindpaw in at least four of five applications. The first step in the Von Frey method is to estimate the response of a filament close to the 50% withdrawal threshold (Chiu et al., 2023b). If no response was observed, a filament with a greater force was used; conversely, if the response was positive, a filament with a lower force was used. This process was continued until four readings following the first crossover response were recorded. Although the responses were near the threshold, the calculation detected a 50% threshold.

### 2.5 Histological staining and cross-sectional area (CSA) analysis

After euthanasia on day 14, the ipsilateral vastus lateralis, biceps femoris, and gastrocnemius muscles were carefully excised, fixed in 10% neutral-buffered formalin for 48 h, and then dehydrated and embedded in paraffin. Longitudinal sections (as shown in Figures 3A–C) with a thickness of 5 μm were cut using a microtome and mounted on glass slides. Sections were stained with hematoxylin and eosin (H&E) according to standard protocols. Briefly, slides were deparaffinized, rehydrated, stained with hematoxylin for nuclear visualization, followed by eosin for cytoplasmic contrast, then dehydrated and mounted.

For cross-sectional area (CSA) analysis, digitized H&E-stained images were captured using a light microscope (Leica) at ×200 magnification. CSA was quantified using ImageJ software. At least eight randomly selected non-overlapping fields per muscle section were analyzed per animal, and individual muscle fiber cross-sectional areas (in arbitrary units, a.u.) were measured manually by outlining fibers using the polygon selection tool. A minimum of 100 fibers per muscle per animal were included in the final analysis.

### 2.6 Transmission electron microscopic (TEM)

TEM was used to visualize cellular structures. A JEOL JEM-1400 electron microscope operating at 120 keV and equipped with an Ultrascan CCD Camera System (Gatan) was used. To ensure unbiased morphometric analysis of organelles, rat cells were experimentally manipulated and fixed by a single researcher. Another researcher who was blinded to the experimental conditions processed and collected images randomly using an electron microscope. Quantitative analysis was performed on anonymized samples by a third researcher who received random images of whole cells at all magnifications.

Mitochondria were counted using the ImageJ software (version 1.54, National Institutes of Health, United States). Healthy mitochondria were characterized by clear, uniform cristae, and intact, distinct outer and inner membranes. Damaged mitochondria were identified as those with uneven or absent cristae, ruptured outer membranes, or significant swelling. Mitochondrial content was measured from images at ×10,000 magnification and expressed as the mitochondrial count per field. For the statistical analysis, data from 20 microscopic photographs of the control, CCI, and CCI + FSN groups were included.

Glycogen storage was quantified manually in each photograph and defined as the number of glycogen granules observed in the TEM field of view. Researchers blinded to the group assignments captured TEM images at ×10,000 magnification across eight different fields of view and manually counted the glycogen granules. The density of glycogen granules (granules/μm^2^) was measured and statistically compared between groups.

### 2.7 RNA extraction and library preparation

For RNA extraction of the sciatic nerves from rats, tissues were frozen at–80°C, and total RNA was extracted using 1 mL of REzolTM C&T reagent (PROtech Technologies, Inc., Taipei, Taiwan, R.O.C.). The phenol–chloroform phase was used for protein isolation according to the manufacturer’s instructions. RNA was extracted using an RNeasy Plus Universal Kit (Qiagen). Library preparation, RNA sequencing, and bioinformatic analyses were performed at the Functional Genomics Center, Zurich. Libraries were prepared using the TruSeq RNA Stranded Library Prep Kit (Illumina, Inc., San Diego, CA, United States), and sequencing was performed usingan Illumina NovaSeq 6000 instrument with 150 bp paired-end reads.

### 2.8 RNA sequencing data analysis

For all samples, the read quality was assessed using FastQC. The data generated from the sequencer were passed through the bcl2fastq conversion program to convert the binary base calls into a common FASTQ format. This FASTQ file is referred to as the raw data. Subsequently, low-quality bases, adapters, and reads containing many unknown N bases were removed from the raw data using FASTP to obtain clean reads. To remove RNA, the reads that filtered out rRNA from the reference genome were compared. Genes with a false discovery rate of <0.05 were defined as differentially expressed, and both log2 fold change and p < 0.01 were used to perform a series of analyses, such as gene ontology (GO), gene set enrichment analysis (GSEA), and function enrichment.

### 2.9 Western blot

Western blotting was performed as previously described (Liu et al., 2022). Briefly, total protein was extracted from cells using RIPA buffer supplemented with protease and phosphatase inhibitors. Protein concentrations were determined by BCA assay, and equal amounts of protein were separated by SDS-PAGE and transferred onto PVDF membranes. Membranes were blocked with 5% non-fat milk and incubated with primary antibodies overnight at 4°C, followed by incubation with appropriate HRP-conjugated secondary antibodies. The bands were visualized using enhanced chemiluminescence (ECL). Details of the antibodies used are provided in Table 1.

**TABLE 1:** Additional antibodies used in the study.

Name	Sp. (clone number or code number)		In the work usage	Vendor
Primary antibody				
FGFR1	ab824	Monoclonal IgG1	WB	Abcam
FGFR3	sc-13121	Mouse monoclonal IgG2a κ	WB	Santa Cruz
HSP60	MA3-012	Monoclonal IgG2a	WB	Invitrogen
PINK1	sc-517353	Mouse monoclonal IgG1 κ	WB	Santa Cruz
VDAC	PA1-954A	Rabbitpolyclonal IgG	WB	Invitrogen
BNIP3L	GTX-636515	Rabbit monoclonal IgG	WB	GeneTex
p-FOXO3	ab47285	Rabbit Polyclonal IgG	WB	Abcam
p-ERK	sc-7383	mouse monoclonal IgG2a κ	WB	Santa Cruz
β-Actin	IR2-7	Rabbit polyclonal IgG	WB	Ireal
Secondary antibody				
m-IgGκ BP-HRP	sc-516102	Mouse IgG kappa binding protein conjugated to HRP	WB	Santa Cruz
Goat anti-rabbit IgG	ab97051	Goat anti-rabbit antibody conjugated to HRP	WB	Abcam

IgG, immunoglobulin G; WB, Western blotting; HRP, horseradish peroxidase.

### 2.10 Detection of the adenosine diphosphate (ADP)/adenosine triphosphate (ATP) ratio, adenosine monophosphate (AMP), and adenosine

The ADP/ATP ratio was calculated using the ApoSENSOR™ ADP/ATP ratio bioluminescence assay kit (cat. no. K255-200), and anadenosine assay kit (cat. no. K327) was purchased from BioVision, Inc. Fresh muscle tissue samples from the vastus lateralis, biceps femoris, and gastrocnemius were collected, and single-cell suspensions were prepared using a 70-μm sieve to confirm cell counts. Briefly, the ADP/ATP ratio assay required 1 × 10^6 cells to be added to a 96-well luminescence plate along with a nucleotide-releasing buffer, following the manufacturer’s instructions. The ADP/ATP ratio was calculated using the formula: ADP/ATP ratio = (C−B)/A.As described above, 10 μL of undiluted muscle sample was mixed with the adenosine assay buffer for adenosine detection, following the manufacturer’s instructions. The sample adenosine concentration (C) was calculated using the formula: C = B/V × D pmol/μ, and the result was normalized to protein concentration to obtain the final measurement. An AMP assay kit (ab273275) was purchased from Abcam. Muscle tissue samples were processed at a protein concentration of 10 mg, as specified by the manufacturer.

### 2.11 Detection of skeletal muscle of growth factors

Skeletal muscle tissue was quickly cryopreserved after isolation, and enzyme-linked immunosorbent assay (ELISA) was performed using a commercially available ELISA kitswith FGF (MFB00, R&D Systems), VEGF (RAB0512, Sigma), NGF (MBS2709258, MyBioSource), and IGF (MG100, R&D Systems). According to the manufacturer’s instructions, the optical density was measured at 450 nm usinga plate reader, and the sample values were calculated from the standard curve.

### 2.12 Transcriptomic dataset analysis

Publicly available gene expression data were retrieved from the Gene Expression Omnibus (GEO) database (https://www.ncbi.nlm.nih.gov/geo/) under the accession number GSE9103. This dataset contains transcriptomic profiles of vastus lateralis muscle biopsies obtained from young and elderly individuals, categorized into sedentary and endurance-trained groups. The following genes of interest were analyzed based on their relevance to mitochondrial autophagy and muscle regeneration: PARK2, BNIP3L, VDAC1, VDAC2, FGFR1, FGFR3. Comparisons were made among the four groups: sedentary young, trained young, sedentary old, and trained old.

### 2.13 Statistical analysis

Statistical analyses were performed as previously described (Cheng et al., 2023). Briefly, all data are presented as means ± standard deviations (SD). Statistical comparisons among three groups were conducted using one-way analysis of variance (ANOVA), followed by Tukey’s post-hoc test to determine intergroup differences. A p-value of less than 0.05 was considered statistically significant. GraphPad Prism version (version 7.0) was used for all statistical analyses and graph generation.

## 3 Results

### 3.1 Impact of FSN treatment on muscle mass and mechanical withdrawal threshold in the CCI rat model

**FIGURE 1:**
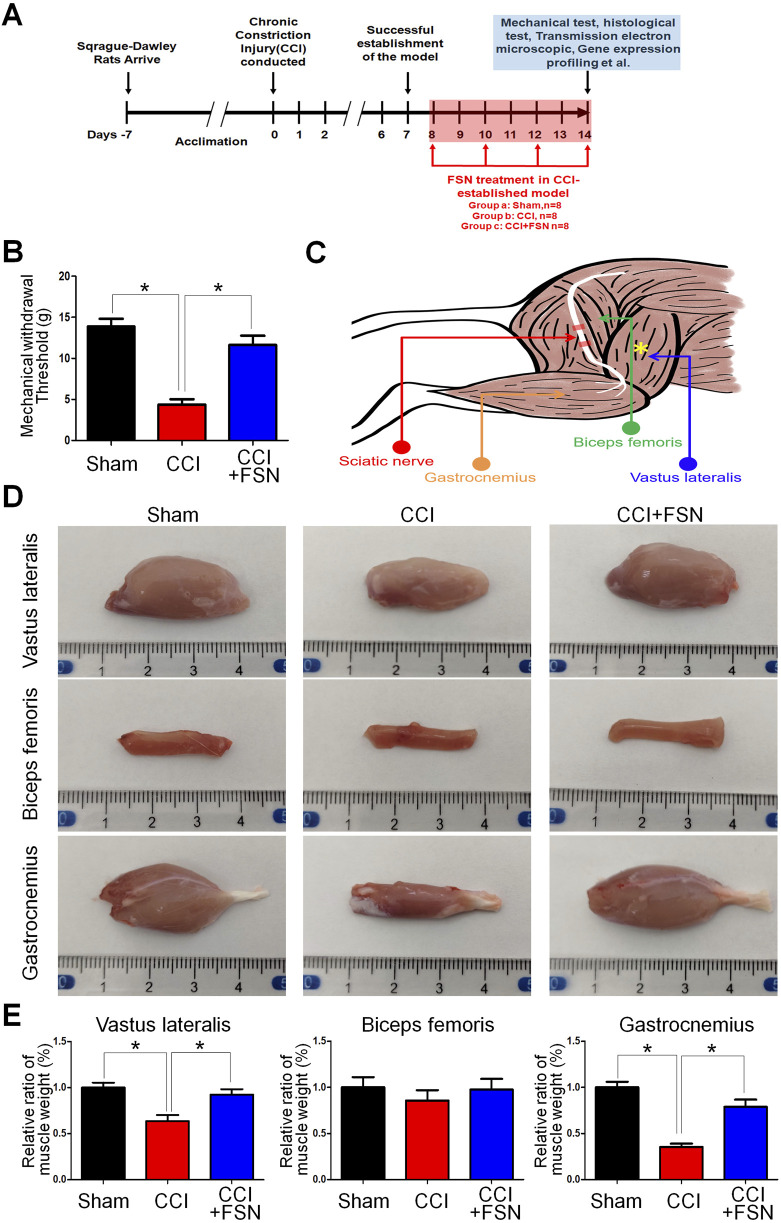
Impact of Fu’s subcutaneous needling (FSN)on muscle mass and mechanical withdrawal threshold in a chronic constriction injury (CCI) rat model. **(A)** Experimental timeline: Sprague–Dawley rats were acclimated for 7 days before inducing the CCI procedure on day 0. Successful establishment of the CCI model was confirmed by day 7. FSN treatment was initiated on day 7 and continued through day 14. Mechanical, histological, and molecular analyses were performed during the final assessment phase (days 11–14). **(B)** Mechanical withdrawal threshold: Bar graph comparing withdrawal thresholds (in grams) between the sham, CCI, and CCI + FSN groups (n = 8 per group). Data indicate a significant reduction in withdrawal thresholds following CCI, which was partially reversed by FSN treatment. **(C)** Anatomical schematic: Illustration of the sciatic nerve and associated muscles, highlighting the vastus lateralis, biceps femoris, and gastrocnemius muscles. These muscles were assessed for structural and weight changes. **(D)**Representative images of dissected vastus lateralis, biceps femoris, and gastrocnemius muscles from the sham, CCI, and CCI + FSN groups. Muscle atrophy was evident in the CCI group, particularly in the vastus lateralis and gastrocnemius, with recovery observed in the CCI + FSN group. **(E)** Relative muscle weight: Bar graphs displaying the percentage ratio of muscle weight to body weight for the vastus lateralis, biceps femoris, and gastrocnemius muscles. Significant reductions in the relative weight of the vastus lateralis and gastrocnemius were observed in the CCI group, which were reversed by FSN treatment. Data are presented as means ± standard errors of the mean (n = 8), *p < 0.01.

To investigate the effects of FSN on muscle mass and mechanical withdrawal thresholds in the CCI rat model, we first established a timeline for the experimental procedures (Figure 1A). After acclimation, the rats underwent the CCI procedure on day 0, with FSN treatment initiated on day 7 and continued until day 14. Mechanical, histological, and molecular analyses were performed during the final assessment phase (days 11–14). The mechanical withdrawal threshold, a measure of neuropathic pain, was significantly lower in the CCI group than in the sham group, indicating successful establishment of the CCI model. FSN treatment partially restored the withdrawal threshold (Figure 1B). The anatomical diagram (Figure 1C) illustrates the relationship between the sciatic nerve and its associated muscles. Representative images of the vastus lateralis, biceps femoris, and gastrocnemius muscles showed marked atrophy in the CCI group, which was alleviated by FSN treatment (Figure 1D). Quantitative analysis revealed a significant reduction in the relative weights of the vastus lateralis and gastrocnemius muscles in the CCI group compared to those in the sham group, which was reversed by FSN treatment (Figure 1E). These findings demonstrated the protective effects of FSN against CCI-induced muscle atrophy and neuropathic pain.

### 3.2 Histological evaluation and cross-sectional area (CSA) analysis of muscles

**FIGURE 2:**
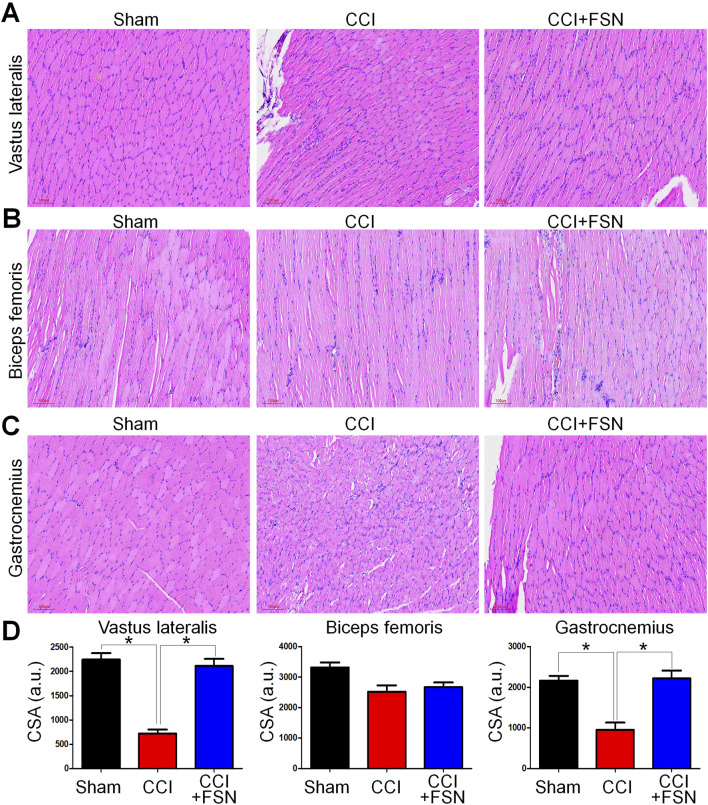
Histological evaluation and cross-sectional area (CSA) analysis of muscles in the sham, chronic constriction injury (CCI), and CCI + Fu’s subcutaneous needling (FSN) groups. **(A–C)** Representative hematoxylin and eosin (H&E) staining images of the vastus lateralis **(A)**, biceps femoris **(B)**, and gastrocnemius **(C)** muscles from the sham, CCI, and CCI + FSN groups. Muscle fibers, especially in the vastus lateralis and gastrocnemius, from the CCI group show signs of atrophy and disorganization compared to those in the sham group. Recovery of muscle structure was observed in the CCI + FSN group. **(D)** Quantitative analysis of muscle fiber cross-sectional area (CSA) in arbitrary units (a.u.) for the vastus lateralis, biceps femoris, and gastrocnemius muscles. A significant reduction in CSA was detected in the CCI group, which was mitigated by FSN treatment. Data are presented as means ± standard errors of the mean (n = 8), *p < 0.01.

Histological analysis using hematoxylin and eosin staining revealed distinct morphological changes in muscle fibers across groups (Figures 2A–C). The vastus lateralis and gastrocnemius muscles in the CCI group displayed significant atrophy and disorganization of muscle fibers compared to those in the sham group. Treatment with FSN ameliorates these structural abnormalities. Quantitative CSA analysis supported these findings, showing significant reductions in CSA in the vastus lateralis and gastrocnemius muscles in the CCI group compared to the sham group, while biceps femoris no significantly change (Figure 2D). FSN treatment restored the CSA to levels comparable to those in the sham group. Thus, FSN treatment may preservethe structure and integrity of CCI-affected muscles.

### 3.3 Ultrastructural changes in muscle fibers and quantitative analysis

**FIGURE 3:**
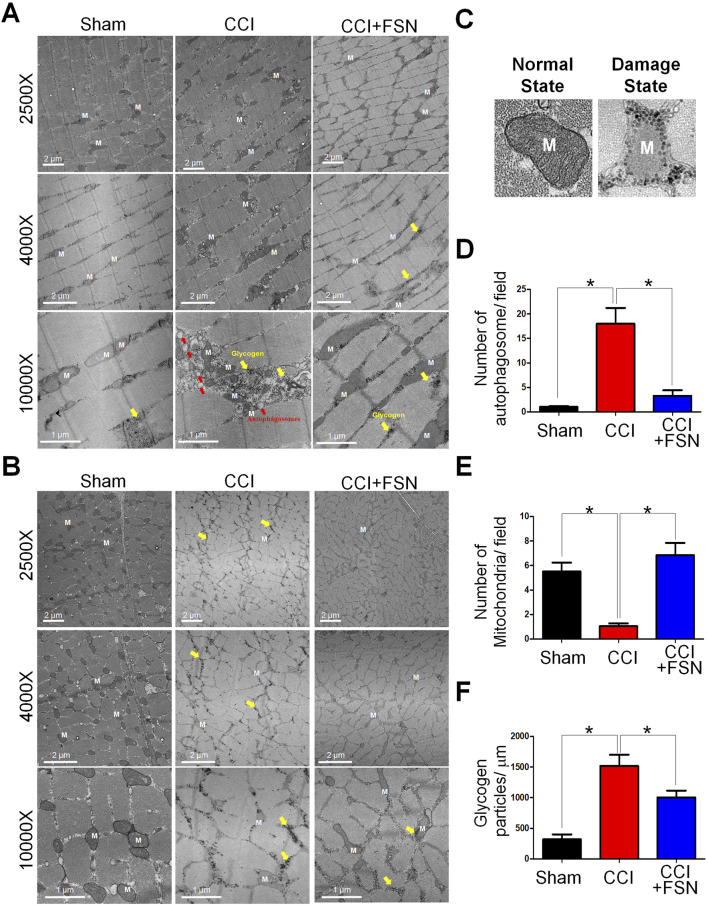
Ultrastructural changes in muscle fibers and associated quantitative analysis in thesham, chronic constriction injury (CCI), and CCI + Fu’s subcutaneous needling (FSN) groups. **(A,B)** Transmission electron microscopy (TEM) images of muscle fibers from the vastus lateralis. The longitudinal section **(A)** and transverse section **(B)** muscles. 2500× and 4000× magnifications: Muscle fibers in the CCI group exhibit mitochondrial (M) damage, including swelling and vacuolation (yellow arrows). FSN treatment restored mitochondrial integrity. ×10,000 magnification: The CCI group shows accumulation of autophagosomes (red arrows) and disrupted glycogen storage (yellow highlights), whereas FSN treatment reduced autophagosome formation and improved glycogen preservation. **(C)** Representative TEM images of mitochondria in a normal state (left) and a damaged state (right), highlighting the structural differences. **(D–F)**Quantitative analysis of muscle ultrastructure: **(D)**Number of autophagosomes per field: Increased autophagosome formation was observed in the CCI group, which was significantly reduced by FSN treatment. **(E)**Number of mitochondria per field: Mitochondrial density was significantly reduced in the CCI group but restored in the CCI + FSN group. **(F)**Glycogen particle density (particles/μm^2^): The CCI group exhibited significantly lower glycogen content than the sham group, whereas FSN treatment markedly improved glycogen preservation. Data are presented as means ± standard errors of the mean (n = 8), *p < 0.01.

Evaluation of mitochondrial status in the vastus lateralis based on the action site of FSN using transmission electron microscopy (TEM). TEM reveals ultrastructural changes in the longitudinal section (Figure 3A) and transverse section (Figure 3B) of vastus lateralis muscle fibers. At lower magnifications, the CCI group exhibited mitochondrial damage including swelling and vacuolation, which was absent in the sham group. Higher magnification highlighted the accumulation of autophagosomes and disrupted glycogen storage in the CCI group (Figure 3C). Treatment with FSN mitigated these changes, restored mitochondrial integrity, and reduced autophagosome formation. The quantitative analysis further supported these observations. The number of autophagosomes per field was significantly increased in the CCI group compared to that in the sham group and was reduced by FSN treatment (Figure 3D). Although mitochondrial density was significantly lower in the CCI group than in the sham group, this recovered with FSN treatment (Figure 3E). Glycogen particle density also decreased in the CCI group and improved with FSN treatment (Figure 3F). Thus, FSN may protect against CCI-induced mitochondrial dysfunction and promote glycogen preservation.

### 3.4 Differential gene expression and pathway enrichment analysis

**FIGURE 4:**
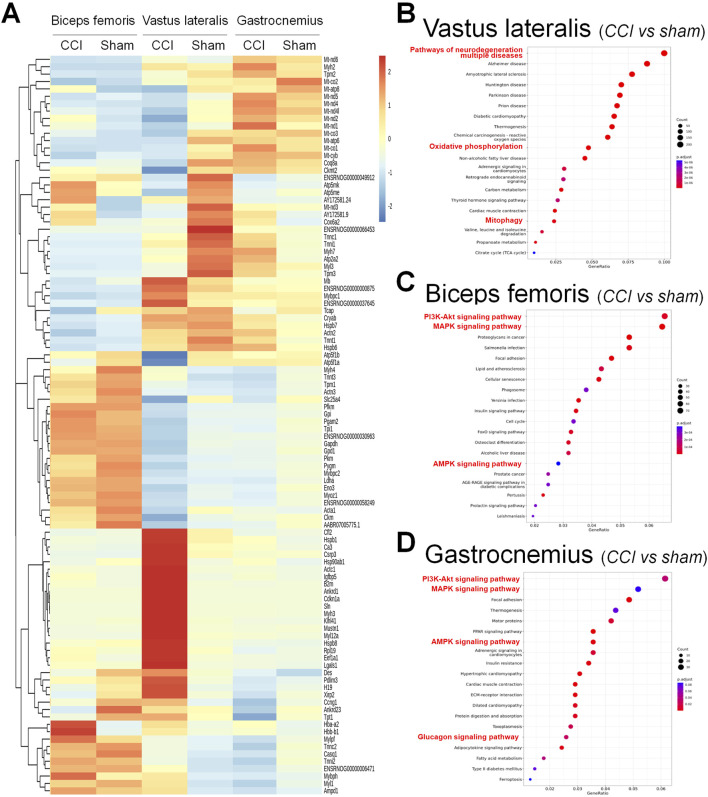
Differential gene expression and pathway enrichment analysis of muscle tissues in the chronic constriction injury (CCI) versus sham conditions. **(A)** Heatmap showing the differential expression of genes across three muscle groups: biceps femoris, vastus lateralis, and gastrocnemius under CCI and sham conditions. The color gradient represents gene expression levels, with red indicating upregulation and blue indicating downregulation. **(B–D)** Pathway enrichment analysis for each muscle type, comparing CCI versus sham. **(B)** Vastus lateralis: Highlighted pathways include neurodegeneration, oxidative phosphorylation, and mitophagy. **(C)** Biceps femoris: Enriched pathways include the PI3K–Akt signaling pathway, MAPK signaling pathway, and AMPK signaling pathway. **(D)** Gastrocnemius: Similar to the biceps femoris, enriched pathways include the PI3K–Akt, MAPK, AMPK, and glucagon signaling pathways. The dot plots display the adjusted p-values and pathway significance, with dot size representing gene count and color scale indicating statistical significance.

RNA sequencing analysis was performed on three muscle groups on the nerve-ligated side 2 weeks after the completion of the CCI ligation surgery. Differential gene expression analysis identified significant transcriptional changes in the biceps femoris, vastus lateralis, and gastrocnemius muscles under CCI conditions compared to those in the sham group (Figure 4A), and volcano plot as showed in the Supplementary Figures S1A-C. Pathway enrichment analysis highlighted pathways related to neurodegeneration, oxidative phosphorylation, and mitophagy in the vastus lateralis muscle (Figure 4B). These results suggest altered mitochondrial function and energy metabolism in response to CCI. Similar pathways including PI3K–Akt, MAPK, and AMPK signalingwere enriched in the biceps femoris and gastrocnemius muscles (Figures 4C,D). These pathways reflect the key biological processes affected by CCI including energy metabolism, stress response, and muscle repair. These findings underscore the systemic effect of CCI on muscle tissues and the potential for targeted interventions to mitigate these effects.

### 3.5 Functional enrichment analysis in the vastus lateralis muscle

**FIGURE 5:**
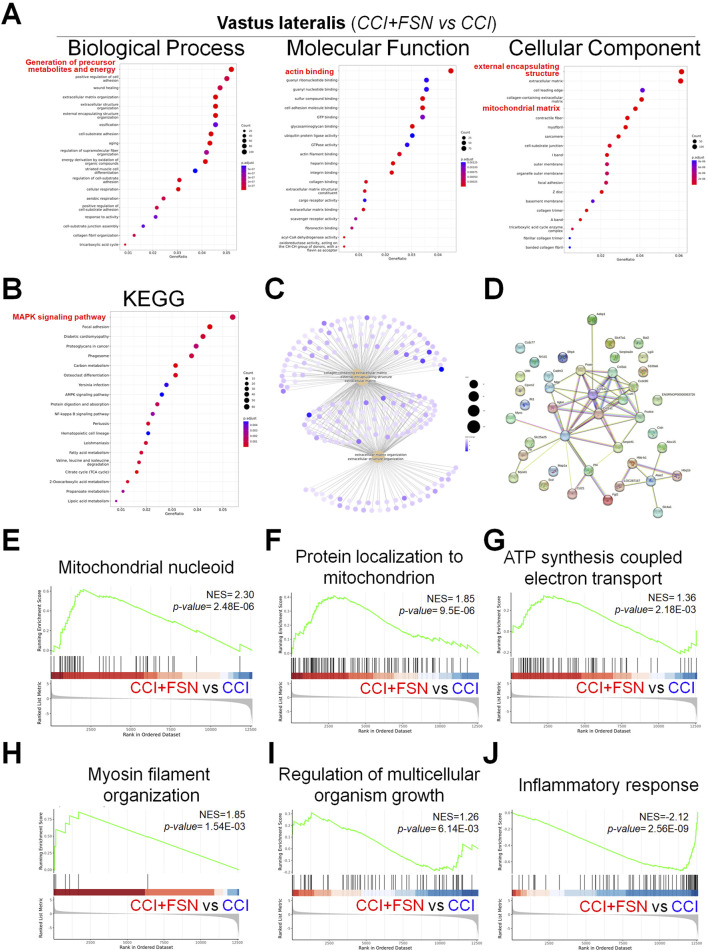
Functional enrichment analysis and gene set enrichment results in the vastus lateralis muscle comparing chronic constriction injury (CCI) +Fu’s subcutaneous needling (FSN) (functional stimulation) versus chronic constriction injury. **(A)** Gene ontology enrichment analysis for biological process, molecular function, and Cellular component categories. The dot plots display enriched terms, with dot size indicating gene count and color representing statistical significance (adjusted p-value). **(B)** Kyoto Encyclopedia of Genes and Genomespathway enrichment analysis shows significant pathwaysincluding MAPK signaling, oxidative phosphorylation, and lipid metabolism pathways. **(C)** Gene-concept network showing associations between enriched biological processes and genes, highlighting key functional modules. **(D)** Protein–protein interaction network for significantly enriched genes, emphasizing their interconnectivity and functional relationships. **(E–J)** Gene set enrichment analysis results highlighting key biological processes and pathways. **(E)** Mitochondrial nucleoid organization (NES = 2.30, p = 2.48E−06). **(F)** Protein localization to mitochondria (NES = 1.85, p = 9.5E−06). **(G)** ATP synthesis coupled electron transport (NES = 1.36, p = 2.18E−03). **(H)** Myosin filament organization (NES = 1.85, p = 1.54E−03). **(I)** Regulation of multicellular organism growth (NES = 1.26, p = 6.14E−03). **(J)** Inflammatory response (NES = −2.12, p = 2.56E-09). Enrichment scores (NES) and p-values demonstrate the significance of functional stimulation (FSN) in modulating specific processes and pathways compared to CCI. Data are presented as means ± standard errors of the mean (n = 3), p < 0.01 is considered statistically significant.

To elucidate the potential molecular mechanisms of FSN treatment for CCI, further RNA sequencing analysis was conducted on the three muscle groups in the CCI + FSN and CCI groups. However, we found no significant differences in the biceps femoris between the CCI + FSN and CCI groups. In contrast, significant differences were observed in the vastus lateralis and gastrocnemius muscles, and principal component analysis as showed in the Supplementary Figures S2A. Significant differences genes as showed in the Supplementary Figures S1B,C. For the vastus lateralis, functional molecular signals by CCI + FSN vs CCI resulted in significant changes in various biological processes, molecular functions, and cellular components, as identified through GO enrichment analysis (Figure 5A). Key enriched biological processes included mitochondrial organization and inflammatory response, whereas molecular functions, such as actin cytoskeleton binding and ATPase activity were significantly modulated. Cellular component analysis highlighted mitochondrial-related structures and extracellular matrix components. Kyoto Encyclopedia of Genes and Genomes (KEGG) pathway enrichment analysis (Figure 5B) revealed the significant involvement of pathways such as oxidative phosphorylation, MAPK signaling, and lipid metabolism. The gene concept network (Figure 5C) demonstrated key associations between the enriched biological processes and genes, identifying functional modules prominently influenced by FSN. Additionally, the protein–protein interaction (PPI) network (Figure 5D) emphasized the interconnectivity and functional relationships among the enriched genes. GSEA further supported these findings, showcasing notable biological processes modulated by FSN (Figures 5E–J). Specifically, mitochondrial nucleoid organization (NES = 2.30, p = 2.48E−06) and protein localization in the mitochondria (NES = 1.85, p = 9.5E−06) were upregulated, indicating enhanced mitochondrial activity. Similarly, ATP synthesis-coupled electron transport (NES = 1.36, p = 2.18E−03) and myosin filament organization (NES = 1.85, p = 1.54E−03) were significantly enriched. Conversely, inflammatory response (NES = −2.12, p = 2.56E−09) was downregulated, suggesting an anti-inflammatory effect of FSN.

### 3.6 Functional enrichment analysis in the gastrocnemius muscle

**FIGURE 6:**
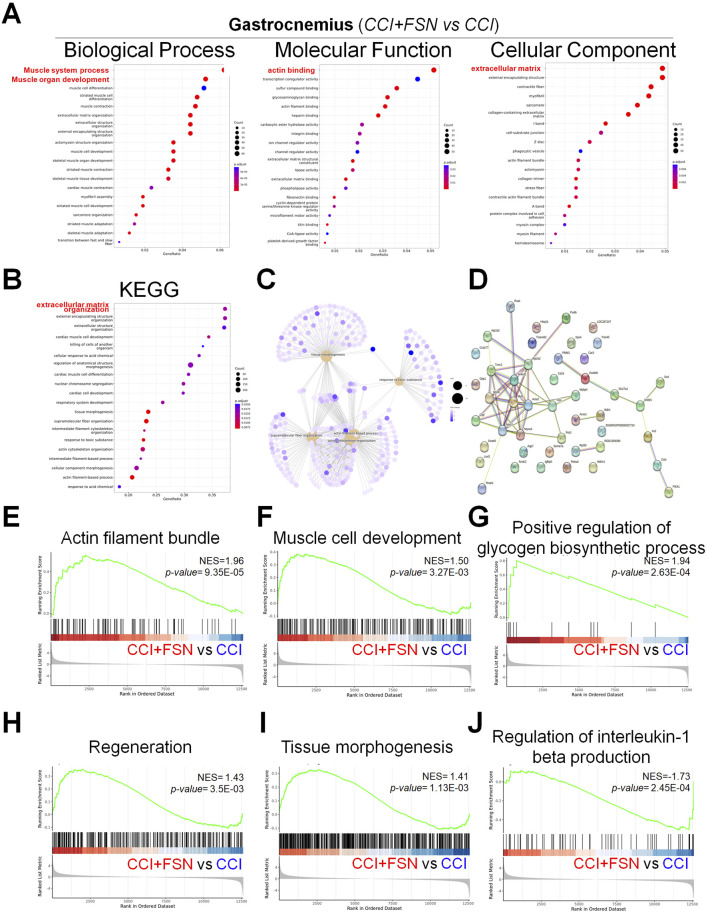
Functional enrichment analysis and gene set enrichment results in the gastrocnemius muscle comparing chronic constriction injury (CCI)+Fu’s subcutaneous needling (FSN) (functional stimulation) versus CCI (chronic constriction injury). **(A)** Gene ontology enrichment analysis for biological process, molecular function, and cellular component categories. The dot plots display enriched terms, with dot size indicating gene count and color representing statistical significance (adjusted p-value). **(B)** Kyoto Encyclopedia of Genes and Genomespathway enrichment analysis, highlighting significant pathways such as extracellular matrix organization, cell adhesion, and oxidative phosphorylation. **(C)** Gene-concept network depicting associations between enriched biological processes and genes, clustering key functional modules. **(D)** Protein–protein interaction network of significantly enriched genes, illustrating their functional relationships. **(E–J)** Gene set enrichment analysis results, demonstrating key biological processes and pathways modulated by FSN: **(E)** Actin filament bundle (NES = 1.96, p = 9.35E−05). **(F)** Muscle cell development (NES = 1.50, p = 3.27E−03). **(G)** Positive regulation of glycogen biosynthetic process (NES = 1.94, p = 2.63E−04). **(H)** Regeneration (NES = 1.43, p = 3.55E−03). **(I)** Tissue morphogenesis (NES = 1.41, p = 1.13E−03). **(J)** Regulation of interleukin-1 beta production (NES = −1.73, p = 2.45E−04). The enrichment scores (NES) and p-values indicate the significant impact of FSN on processes, such as muscle regeneration, energy metabolism, and inflammation. Data are presented as means ± standard errors of the mean (n = 3), p < 0.01 is considered statistically significant.

In the gastrocnemius muscle, FSN also led to notable changes in biological processes, molecular functions, and cellular components (Figure 6A). Biological processes, such as muscle organ development and extracellular matrix organization, were enriched. Molecular functions including the structural constituents of muscle and actin binding were modulated, whereas cellular component analysis highlighted collagen-containing extracellular matrix and contractile fibers. KEGG pathway analysis (Figure 6B) revealed significant pathways including oxidative phosphorylation, extracellular matrix organization, and cell adhesion. The geneconcept network (Figure 6C) and PPI network (Figure 6D) further highlighted the key functional modules and interconnectivity among the enriched genes. The GSEA results (Figures 6E–J) demonstrated that FSN enhanced biological processes such as actin filament bundle organization (NES = 1.96, p = 9.35E−05), muscle cell development (NES = 1.50, p = 3.27E−03), and regeneration (NES = 1.43, p = 3.55E−03). Additionally, positive regulation of glycogen biosynthetic processes (NES = 1.94, p = 2.63E−04) was observed, indicating improved energy metabolism. Similar to the vastus lateralis, FSN downregulated inflammatory pathways, as evidenced by the regulation of interleukin-1 beta production (NES = −1.73, p = 2.45E−04). These results highlight the significant role of FSN in modulating key biological processes and pathways in both the vastus lateralis and gastrocnemius muscles. FSN enhances mitochondrial function, energy metabolism, and muscle regeneration, while mitigating inflammatory responses, underscoring its therapeutic potential for managing CCI.

### 3.7 Changes in energy metabolism and protein expression following FSN treatment

**FIGURE 7:**
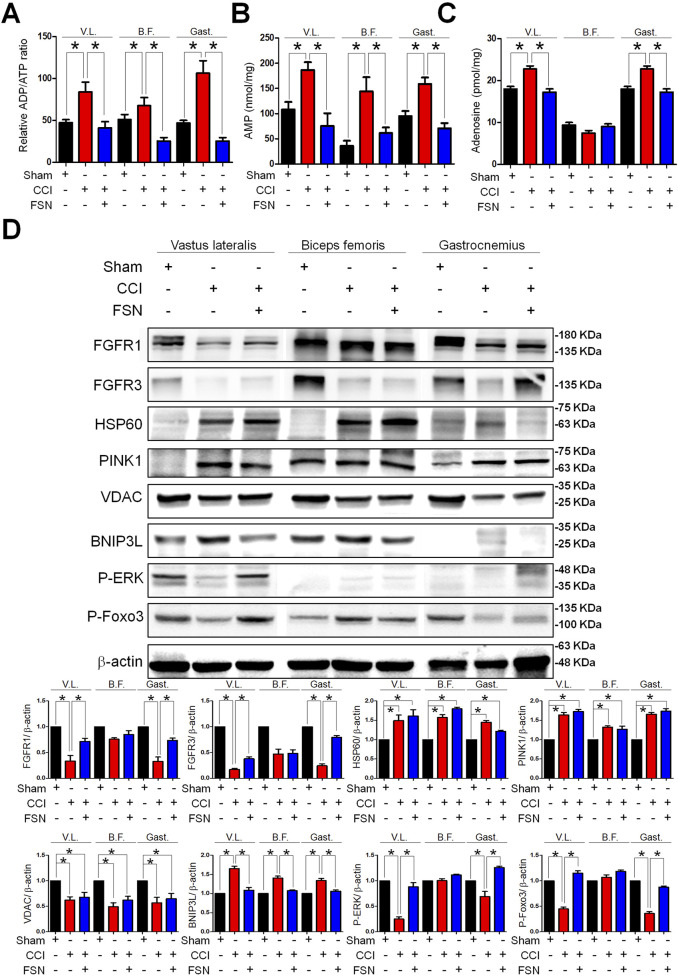
Changes in energy metabolism and protein expression in skeletal muscle following chronic constriction injury (CCI) and Fu’s subcutaneous needling (FSN) treatments. **(A–C)** Quantification of relative adenosine diphosphate (ADP)/adenosine triphosphate (ATP) ratio **(A)**, adenosine monophosphate (AMP) levels **(B)**, and adenosine levels **(C)** in the vastus lateralis (V.L.), biceps femoris (B.F.), and gastrocnemius (Gast.) muscles under different conditions as follows: sham, CCI, and FSN treatment. **(D)** Western blot analysis of key proteins involved in energy metabolism and stress response including FGFR1, FGFR3, HSP60, PINK1, VDAC, BNIP3L, phosphorylation-ERK, and phosphorylation-FOXO3 across the vastus lateralis, biceps femoris, and gastrocnemius muscles. β-Actin is shown as the loading control. The experimental groups included the sham, CCI, and FSN-treated groups. Data are presented as means ± standard errors of the mean (n = 8). *p < 0.01.

Finally, we evaluated changes in energy metabolism and protein expression across the experimental groups. Quantification of the ADP/ATP ratio, AMP levels, and adenosine levels revealed significant disruptions in energy homeostasis in the CCI group compared to that in the sham group, which was ameliorated by FSN treatment (Figures 7A–C). Meanwhile, in the vastus lateralis and gastrocnemius muscles, FSN treatment significantly increase FGF, NGF and IGF compared to that in the CCI group, but VEGF not significant, showed in the Supplementary Figures S3A-D. Western blot analysis of key proteins involved in energy metabolism and stress responses showed altered expression of FGFR1, FGFR3, HSP60, PINK1, VDAC, BNIP3L, phosphorylation-ERK, and phosphorylation-FOXO3 in the CCI group (Figure 7D). Among them, the most significant changes were observed in the vastus lateralis and gastrocnemius muscles. Compared to the sham group, the CCI group exhibited increased expression of autophagy-related markers HSP60, PINK1, VDAC, and BNIP3L, while showing reduced activation of growth factor-related pathways, including FGFR1, FGFR3, phosphorylation-ERK, and phosphorylation-FOXO3. However, compared to the CCI group, the CCI + FSN group demonstrated a significant reduced BNIP3L expression and an increase FGFR1, FGFR3, phosphorylation-ERK, and phosphorylation-FOXO3, without affecting the expression of HSP60, PINK1, or VDAC. Therefore, FSN treatment restored the expression levels of these proteins toward those observed in the sham group, indicating their role in maintaining mitochondrial function and the cellular stress response. Collectively, these findings demonstrated that FSN treatment effectively mitigated CCI-induced disruptions in muscle energy metabolism and protein expression, highlighting its therapeutic potential in neuropathic conditions.

### 3.8 The expression levels of mitophagy- and growth-related genes were analyzed in vastus lateralis muscle samples obtained from sedentary and trained young and old individuals

**FIGURE 8:**
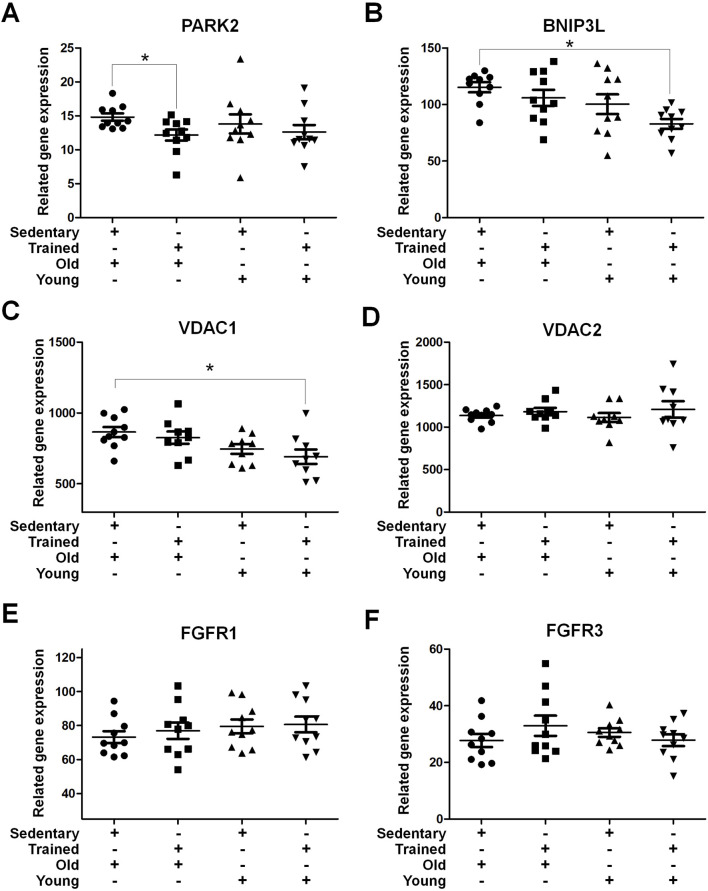
Mitochondrial Autophagy and Growth-Related Receptor Gene Expression in Skeletal Muscle Samples from Sedentary or Trained Young and Elderly Individuals The presented gene expression data were retrieved from the GEO database, specifically from the dataset GSE9103. This dataset contains gene expression profiles of vastus lateralis muscle samples comparing the effects of age and training status. Gene expression analysis of **(A)** PARK2, **(B)** BNIP3L, **(C)** VDAC1, **(D)** VDAC2, **(E)** FGFR1, and **(F)** FGFR3 in vastus lateralis muscle samples from sedentary and trained young and old individuals. The data were normalized and presented as related gene expression. Data are presented as mean ± SEM, *p < 0.05.

Prolonged sedentary lifestyles are a significant factor contributing to nerve compression. To assess the impact of nerve compression on the vastus lateralis muscle, a further analysis of the research database focusing on age and sedentary behavior grouping was conducted. The data for this analysis were obtained from the publicly available GSE9103 dataset. In the mitophagy-related genes, A significant decrease in PARK2 expression was observed in trained old individuals compared to their sedentary counterparts (p < 0.05), no significant differences were observed between the young groups (Figure 8A). The expression of BNIP3L and VDAC1 were significantly upregulated in sedentary older individuals compared to trained young individuals (p < 0.05) (Figures 8B,C). No significant differences were detected in VDAC2 expression across all groups (Figure 8D). Among the growth-related genes, FGFR1 levels in the sedentary elderly group showed a slight decrease compared to the trained young group (73.24 ± 3.464 vs 80.65 ± 4.610; p = 0.215) (Figure 8E). Similarly, FGFR3 demonstrated a comparable trend, with lower levels observed in the sedentary elderly group compared to the trained older group (27.74 ± 2.324 vs 32.93 ± 3.578; p = 0.240). These results suggest that a sedentary lifestyle, one of the contributing factors to sciatica, may influence the expression of specific mitophagy-related genes, such as PARK2 and BNIP3L, which exhibit differential expression in response to sedentarism and aging. In contrast, growth-related genes, such as FGFR1 and FGFR3, are mildly decrease. This finding may have important implications for developing interventions to optimize skeletal muscle health in the context of sciatica.

## 4 Discussion

Sciatic nerve pain, which often results from CCI, is commonly used to study neuropathic pain and its systemic effects. In our study, CCI induced significant atrophy in the vastus lateralis, biceps femoris, and gastrocnemius muscles. Previous studies have demonstrated that prolonged neuropathic pain leads to muscle wasting and structural disorganization owing to reduced motor neuron activity and increased inflammatory signaling (Langer et al., 2018; Yadav and Dabur, 2024). Muscle degradation is exacerbated by oxidative stress and mitochondrial dysfunction, which are the hallmarks of neuropathy-induced muscle damage. Our findings are consistent with those of these reports, showing substantial reductions in muscle weight, cross-sectional area, and mitochondrial density in the CCI group. The preservation of muscle structure and function in FSN-treated animals underscores the potential for targeted interventions to mitigate neuropathy-related muscle atrophy. The FSN technique has gained attention owing to its therapeutic potential in musculoskeletal disorders. Clinical studies have reported that FSN alleviates muscle pain, improves blood flow, and enhances muscle regeneration (Chiu et al., 2024; Wu et al., 2024; Zhang et al., 2024). In animal models, FSN restores muscle fiber integrity, reduces inflammatory cytokine levels, and enhances mitochondrial biogenesis (Li et al., 2022). Our study corroborates these findings, demonstrating that FSN treatment not only improves mechanical withdrawal thresholds but also reverses CCI-induced muscle atrophy and mitochondrial dysfunction. FSN targets both metabolic and structural pathways to support muscle health by enhancing glycogen storage, reducing autophagosome formation, and restoring mitochondrial integrity. These results provide compelling evidence for the use of FSN as an intervention in neuropathic conditions.

Mitochondrial quality control including mitophagy and ATP synthesis is crucial for the maintenance of muscle health. Dysregulated mitophagy has been implicated in various muscle-related diseases including muscular dystrophy and sarcopenia (Romanello and Sandri, 2015; Triolo et al., 2022). Aberrant glucose levels negatively impact neuronal activity and survival (Han et al., 2023). CCI disrupted mitochondrial integrity, reduced mitochondrial density, and impaired glycogen storage, a finding which is consistent with those in previous reports (Li et al., 2022). In particular, FoxO3 and BNIP3-dependent mitophagy may exacerbate skeletal muscle atrophy (Mammucari et al., 2007; Wang et al., 2023). Our results suggested that FSN inhibited mitochondrial autophagy by modulating and attenuating the BNIP3L level. Thus, FSN may be a promising strategy for managing muscle diseases associated with neuropathy by targeting mitochondrial dysfunction. Fibroblast growth factor receptors FGFR1 and FGFR3 are critical mediators of muscle repair and regeneration. These receptors activate downstream PI3K/Akt and MAPK signaling pathways, which are crucial for cell survival, proliferation, and differentiation, and inhibit the Foxo3/BNIP3 autophagy pathway (Chen et al., 2022a; Chen et al., 2022b; Farooq et al., 2021; Neel et al., 2013). Our study identified altered expression levels of FGFR1 and FGFR3 in CCI-affected muscles and showed that FSN treatment restored their expression. These results align with those of previous studies showing that FGFR-mediated activation of PI3K/Akt promotes mitochondrial function and energy metabolism, whereas MAPK signaling regulates myogenic differentiation and stress responses (Zhao et al., 2020). Although FGFR3 may counteract the FGFR1 pathway and potentially promote muscle mass decrease via autophagyin age-related diseases (Li X. R. et al., 2024), our proteomic validation in muscle tissues indicated that both FGFR1 and FGFR3 were significantly downregulated in the CCI animal model. This downregulation may be one of the factors contributing to mitochondrial dysfunction and energy metabolism abnormalities. In the future, it would be valuable to further validate the causal relationships of the involved molecular pathways through gene knockout or chemical activation strategies.

Restoration of these pathways in muscles treated with FSN therapy highlights the mechanistic basis of its therapeutic effects, emphasizing its potential to modulate mitochondria and target FGFR signaling in neuropathy-associated muscle disorders. Emerging evidence suggests that muscle-derived growth factors play critical roles in tissue repair and regeneration. Growth factors such as IGF-1, VEGF, and HGF enhance muscle regeneration and support neural repair (Floriano et al., 2022; Tuffaha and Lee, 2024). Our previous study has highlighted the role of secreted growth factors in promoting axonal growth and reducing neuroinflammation (Chiu et al., 2024). FSN therapy may promote mitochondrial activation through electrical potential responses and enhance growth factor secretion via localized tissue disruption, thus serving as a critical factor in mitigating mitochondrial autophagy and facilitating muscle tissue repair. This aligns with the hypothesis that the growth factors involved in nerve repair during FSN therapy may originate from these processes. In our CCI-induced animal model, the molecular mechanisms of the three muscle parts exhibited significant differences compared to the sham group. However, macroscopically, the vastus lateralis and gastrocnemius muscles showed significant changes in weight and appearance, whereas the biceps femoris did not exhibit notable differences. This may be related to the varying severity of the established animal model. Additionally, regarding FSN treatment, clinical practice often targets the affected site as the treatment area, with the therapeutic effects gradually diminishing as the distance from the affected site increases. Based on these clinical findings, it is reasonable to speculate that the lack of significant differences in RNA sequencing results for the biceps femoris between the CCI + FSN and CCI groups may be attributed to two factors: first, the biceps femoris in our CCI animal model did not display pathological changes as pronounced as those in the vastus lateralis and gastrocnemius; second, the presence of tissue barriers between the FSN treatment site and the affected area may have influenced the therapeutic efficacy. This hypothesis warrants further investigation to elucidate the interplay between muscle and nerve recovery mechanisms. Moreover, among the muscles analyzed, the biceps femoris showed limited improvement in cross-sectional area (CSA) and weight recovery following FSN treatment, compared to the vastus lateralis and gastrocnemius. This discrepancy may be related to the intrinsic composition and distribution of muscle fiber types (Edgerton et al., 1975). The biceps femoris in rodents, particularly in Sprague–Dawley rats, has been reported to contain a higher proportion of Type II (fast-twitch) fibers, which are more glycolytic and prone to atrophy under denervation or disuse conditions. Type I (slow-twitch) fibers, which are more oxidative and fatigue-resistant, may respond more favorably to interventions such as FSN that promote mitochondrial function and energy metabolism (Kovanen et al., 1984). Although our current study did not specifically quantify fiber-type composition, this fiber-type heterogeneity may partly explain the differential responsiveness observed. Future studies incorporating fiber-typing are warranted to elucidate the role of muscle fiber distribution in FSN-mediated recovery.

## 5 Conclusion

**FIGURE 9:**
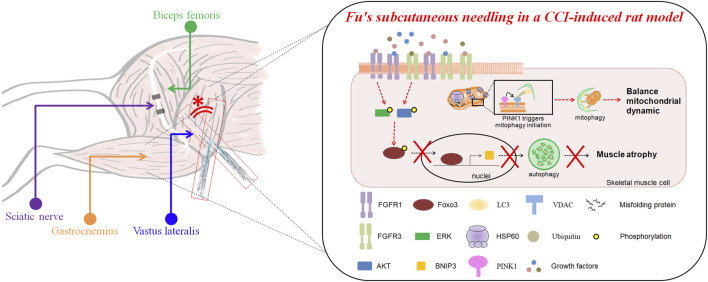
The impact of Fu’s subcutaneous needling on autophagy and muscle mass loss in a CCI-induced mice model. The upper section illustrates the anatomical relationships of the biceps femoris, sciatic nerve, gastrocnemius, and vastus lateralis muscles, with the site of Fu’s subcutaneous needling marked (*). The lower section explains the molecular mechanisms in the CCI (chronic constriction injury)-induced mice model. CCI induces mitochondrial dysfunction, leading to the accumulation of misfolded proteins stimulated HSP60 increase and activation of mitophagy via the PINK1-VDAC-LC3 signaling and FOXO3-BNIP3 pathway. This process ultimately results in muscle mass loss. Fu’s subcutaneous needling inhibits the autophagy pathway though FOXO3-BNIP3 axis, preventing excessive mitochondrial degradation and preserving muscle mass.

FSN treatment effectively mitigated CCI-induced muscle atrophy, mitochondrial dysfunction, and impaired energy metabolism. By restoring FGFR1/FGFR3 signaling and enhancing mitochondrial quality control, FSN supported muscle repair and could potentially facilitate neural recovery. Our novel findings elucidate the role of growth factor signaling-mediated mitochondrial recovery in neuropathic repair and underscore the importance of exploring the interconnections between muscle and nerve pathologies (Figure 9). Future research should aim to identify the specific growth factors involved and their contributions to neuromuscular repair processes, thus paving the way for integrative therapeutic strategies.

## Data Availability

The datasets presented in this study can be found in online repositories. The names of the repository/repositories and accession number(s) can be found in the article/Supplementary Material.

## Abbreviations

FSN, Fu’s subcutaneous needling; CCI, Chronic constriction injury; MTrP, Myofascial trigger point; TEM, Transmission electron microscopy; GOBP, Gene ontology biological process.
