# Supplementary material for: Fu’s subcutaneous needling facilitates muscle repair by regulating mitochondrial homeostasis in rat with chronic peripheral nervous pain
Source: Front Physiol. 2025 Aug 21;16:1640735. doi: 10.3389/fphys.2025.1640735 (PMC12408641; doi:10.3389/fphys.2025.1640735)
Supplement: Supplementary file 1 [file Supplementaryfile1.docx]

***Supplementary Information***

**Fu's subcutaneous needling facilitates muscle repair by regulating mitochondrial homeostasis in rat with chronic peripheral nervous pain**

Po-En Chiu ^a,b*^, Zhonghua Fu^c,d^**^*^**, Hung-Chuan Pan^e,f,g,h^, Yi-Ching Tsai^i^, Chia-Yun Tsai^j^, Wei-Jen Hsu^j^, Li-Wei Chou^k,l,m^, De-Wei Lai^j^

**
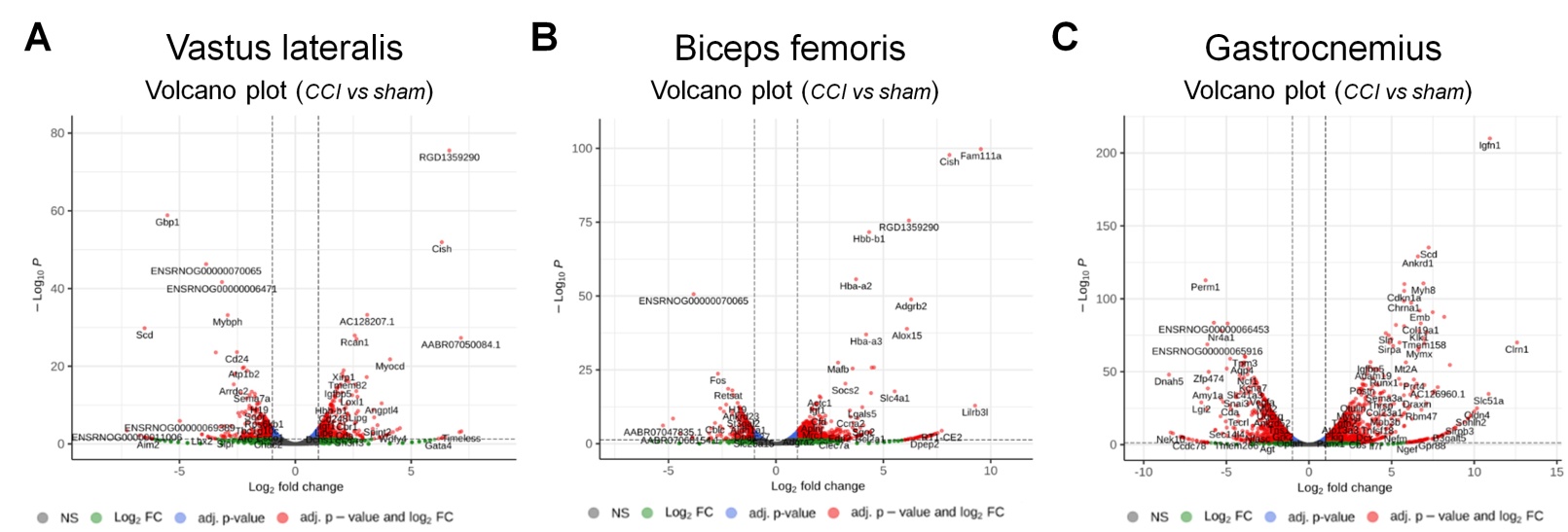
**

**Supplementary Figure 1. Transcriptomic changes in different skeletal muscle types in response to CCI.**

(A–C) Volcano plots showing differential gene expression (CCI vs sham) in the (A) vastus lateralis, (B) biceps femoris, and (C) gastrocnemius muscles. Genes with significant changes in expression are highlighted based on fold change and adjusted p-values: gray dots, not significant (NS); green dots, significant log₂ fold change (FC); blue dots, significant adjusted p-value; red dots, significant for both adjusted p-value and log₂ FC. Dashed vertical lines represent log₂ FC thresholds, and the horizontal dashed line indicates the adjusted p-value threshold.

**
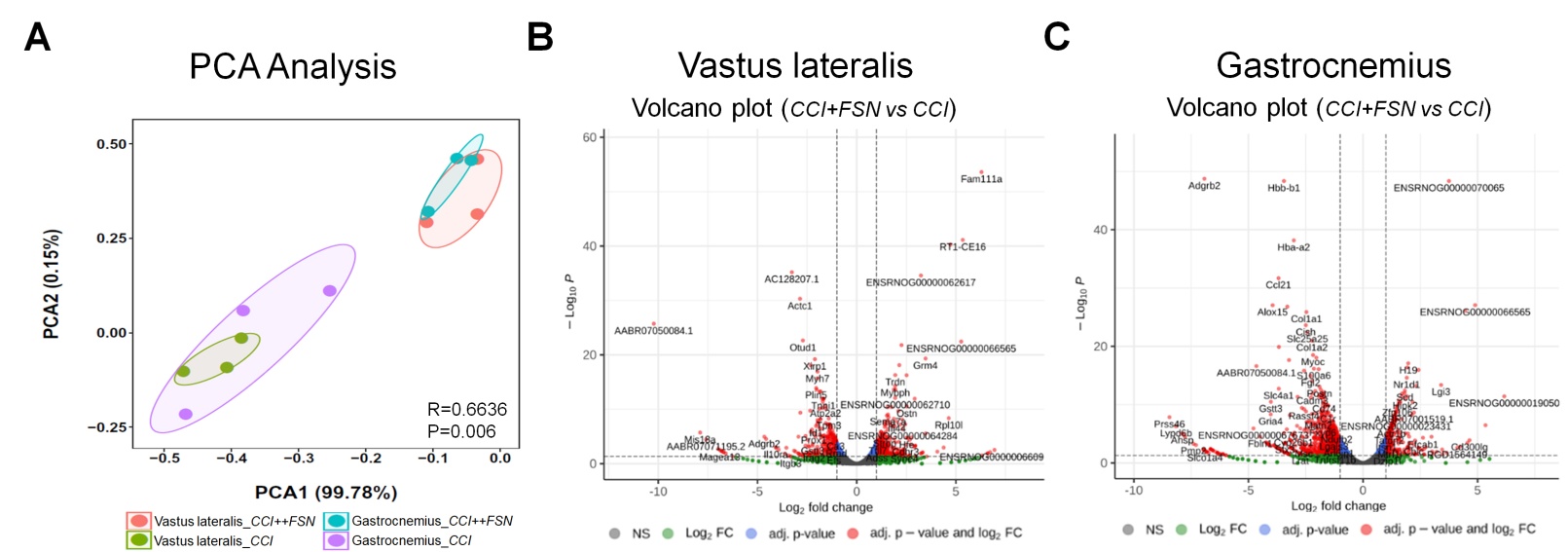
**

**Supplementary Figure 2. Effect of FSN treatment on gene expression in CCI-affected skeletal muscle.**

(A) Principal component analysis (PCA) of gene expression profiles in vastus lateralis and gastrocnemius muscles across CCI and CCI + FSN groups, showing clear separation between treatment groups (R = 0.6636, P = 0.006). (B, C) Volcano plots of differentially expressed genes comparing CCI + FSN vs CCI in (B) vastus lateralis and (C) gastrocnemius muscles. Genes with significant changes in expression are highlighted based on fold change and adjusted p-values: gray dots, not significant (NS); green dots, significant log₂ fold change (FC); blue dots, significant adjusted p-value; red dots, significant for both adjusted p-value and log₂ FC. Dashed vertical lines represent log₂ FC thresholds, and the horizontal dashed line indicates the adjusted p-value threshold.

**
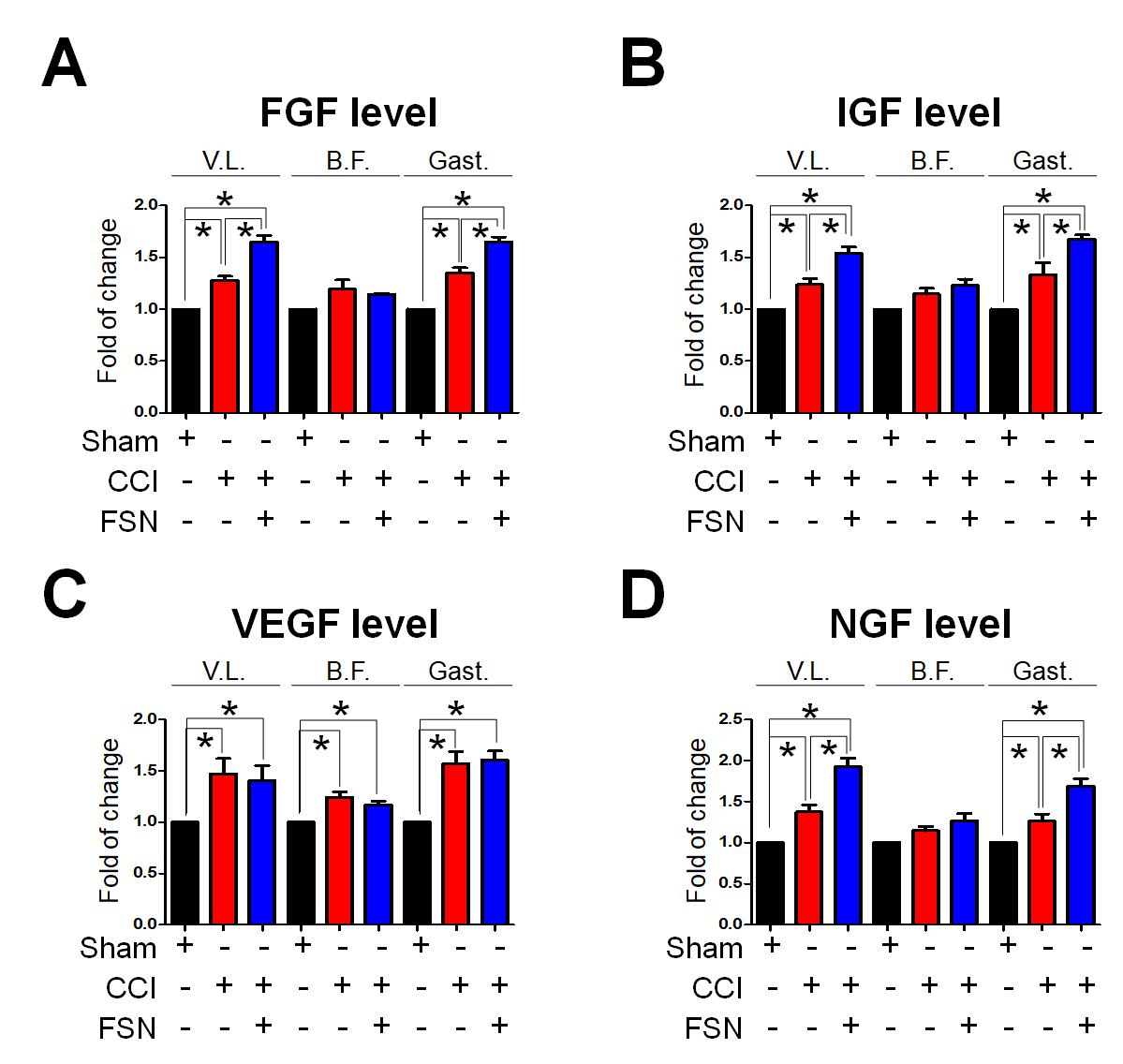
**

**Supplementary Figure 3. Effect of FSN treatment on growth factor levels in skeletal muscles following CCI.**

(A–D) Bar graphs showing fold changes in protein levels of (A) fibroblast growth factor (FGF), (B) insulin-like growth factor (IGF), (C) vascular endothelial growth factor (VEGF), and (D) nerve growth factor (NGF) in the vastus lateralis (V.L.), biceps femoris (B.F.), and gastrocnemius (Gast.) muscles. Groups include sham, CCI, and CCI with FSN treatment. Data are presented as mean ± SEM. Statistical significance was determined by one-way ANOVA with post hoc analysis. **P* < 0.01.
